# Case Report: Bilateral congenital fourth metacarpal shortening in a competitive softball athlete with preserved function

**DOI:** 10.3389/fsurg.2026.1835514

**Published:** 2026-07-08

**Authors:** Sui-Ling Perez, Rakesh R. Nair, Noah S. Llaneras, Emily Garateix, Lidia Sabater, Roberto A. Miki

**Affiliations:** 1 Herbert Wertheim College of Medicine, Florida International University, Miami, FL, United States; 2 Florida International University, Miami, FL, United States

**Keywords:** absent knuckle sign, athletic participation, brachydactyly type E, brachymetacarpia, congenital hand anomaly, fourth metacarpal shortening, hand function, incidental finding

## Abstract

**Background:**

Congenital shortening of the fourth metacarpal (brachydactyly type E) is an uncommon anatomical variant that may occur in isolation or with syndromic conditions. Prior case reports have documented this finding in adults, but none have assessed functional outcomes in the context of high-level athletic participation.

**Case presentation:**

A 67-year-old woman presented to the emergency department on two separate occasions after falling on her left and right wrists. Radiography and computed tomography revealed a non-displaced triquetral fracture (left) and a non-displaced scaphoid fracture (right), with incidental bilateral symmetric shortening of the fourth metacarpals. Radiographic archives spanning more than a decade have confirmed stable morphology, thereby supporting a congenital etiology. The patient reported no history of hand dysfunction and had competed in softball at the high school level and internationally as part of her national team. Following conservative management, the patient regained a full, pain-free range of motion bilaterally.

**Discussion:**

This case is the first report of bilateral congenital shortening of the fourth metacarpal in a lifelong competitive athlete, demonstrating that this variant is compatible with high-level athletic participation without self-reported functional limitations. Radiographic stability for more than a decade and preserved function into late adulthood extend the evidence base for the long-term benignity of this condition. Formal grip dynamometry and validated functional scores were not available, which is acknowledged as a limitation of this study.

**Conclusion:**

Conservative management with reassurance is appropriate for asymptomatic patients. Recognition of this radiographic appearance can prevent misinterpretation as traumatic shortening or growth plate injury.

## 1 Introduction

Congenital shortening of the fourth metacarpal is an uncommon musculoskeletal variant classified as type E brachydactyly, which was first described by Bell (1). It may occur in isolation or as a part of syndromic conditions, including Turner syndrome (2–4), pseudohypoparathyroidism with Albright's hereditary osteodystrophy (5–7), or idiopathic hypoparathyroidism (8, 9). The prevalence of isolated cases is currently unknown. Most patients are asymptomatic, and diagnosis is often incidental or prompted by the “absent knuckle sign” (5). Recognition of its benign nature is important to prevent unnecessary investigations or surgical interventions.

Prior case reports have documented isolated metacarpal shortening in adults, including bilateral involvement in some cases (10–13). However, existing reports have focused primarily on radiographic descriptions, syndromic exclusion, or surgical correction techniques (14), and none have examined functional outcomes in the context of high-level athletic participation. We present a case of bilateral congenital shortening of the fourth metacarpal in a 67-year-old woman, identified incidentally following separate wrist injuries. What distinguishes this case is the patient's history of competitive softball at the high school level and internationally as part of her national team, providing novel evidence that this variant is compatible with sustained high-demand hand use.

## 2 Case description

A 67-year-old woman presented to the emergency department (ED) after a fall on her left wrist. Radiographic imaging and a computed tomography (CT) scan conducted in the ED revealed a shortening of the fourth metacarpal and a suspected triquetral fracture. The CT scan confirmed a non-displaced fracture of the triquetrum (Figure 1).

**Figure 1:**
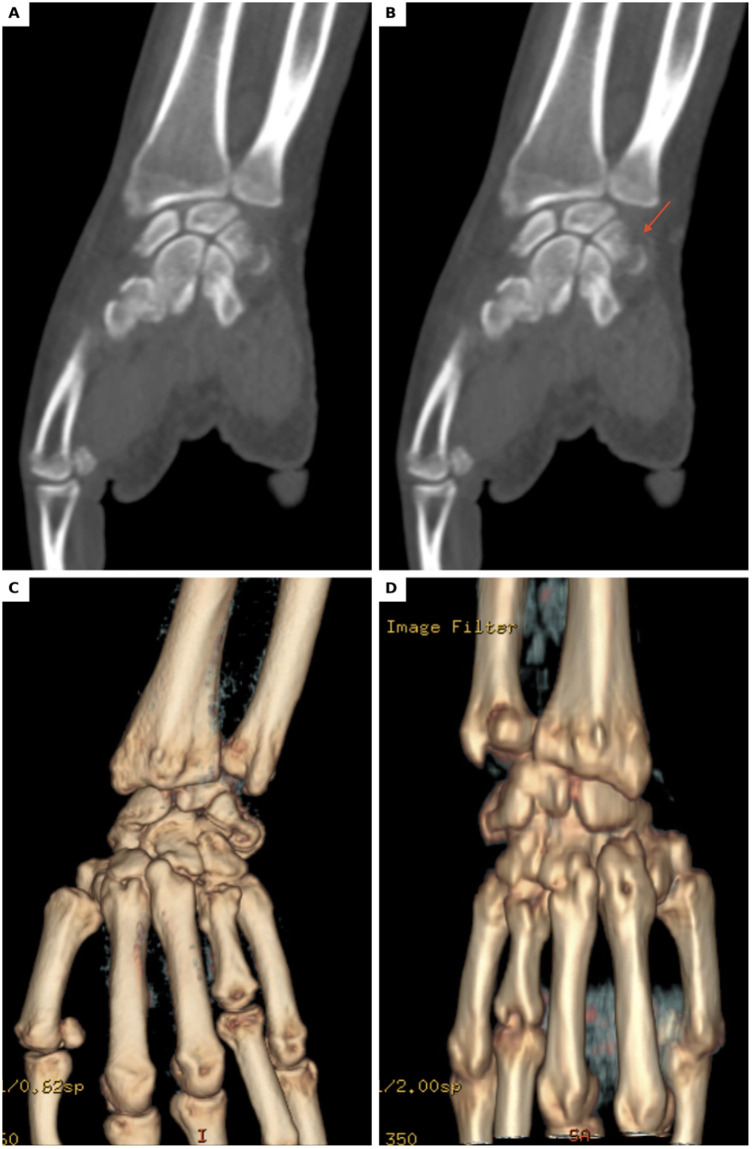
Computed tomography of the left wrist demonstrating the non-displaced triquetral fracture and congenital fourth metacarpal shortening. **(A)** Coronal CT slice of the left wrist without annotation. **(B)** The same coronal projection with a non-displaced triquetral fracture is indicated by the red arrow. **C**) Three-dimensional CT reconstruction of right hand. **D**) Three-dimensional CT reconstruction of left hand. Both images illustrate symmetric diaphyseal shortening of the fourth metacarpal relative to the adjacent third and fifth metacarpals.

Her medical history was significant for type II diabetes mellitus, peripheral artery disease, hypercholesterolemia, and hypertension. She had no history of parathyroid disease. Her medications included metformin, digoxin, enalapril, simvastatin, warfarin, and aspirin. Her surgical history included hysterectomy, pacemaker placement, and right lower extremity amputation. She had no history of tobacco or illicit drug use and no known drug allergies.

Several patient factors are relevant to fall and fracture risk and are unrelated to congenital metacarpal shortening. Type II diabetes mellitus and peripheral artery disease may contribute to peripheral neuropathy, impaired balance, and altered gait, and prior right lower extremity amputation alters gait mechanics and may affect both fall risk and protective upper-extremity use during a fall. Concurrent warfarin and aspirin therapy was also noted. The first fall occurred when the patient tripped while doing laundry and sustained a ground-level mechanical fall from a standing height. The second fall occurred approximately 1 year later when the patient, seated in a wheelchair, became entangled in the wheels by her dress and fell forward onto an outstretched right hand.

The patient presented as an outpatient for further evaluation following the first injury. On physical examination, she was 5'6" tall and weighed 144 lbs. She had tenderness over the left triquetrum and shortening of the left fourth metacarpal, along with swelling and a limited range of motion in the left hand due to pain. Conservative treatment was initiated using a wrist-control splint for 6 weeks. At the 6-week follow-up, the patient regained a full range of motion in all fingers of the left hand.

Approximately 1 year later, the patient sustained a second fall, this time involving the right wrist. Plain radiographs of the right wrist and hand obtained in the ED were negative for fractures; no dedicated scaphoid view was obtained at that time. A CT scan subsequently ordered by another orthopedic surgeon demonstrated an acute non-displaced scaphoid fracture (Figure 2), along with congenital shortening of the fourth metacarpal, mirroring the left hand. The treatment consisted of cast immobilization for three months. A follow-up CT scan confirmed bony union of the scaphoid fracture, and the patient regained a full, pain-free range of motion in both hands and wrists.

**Figure 2:**
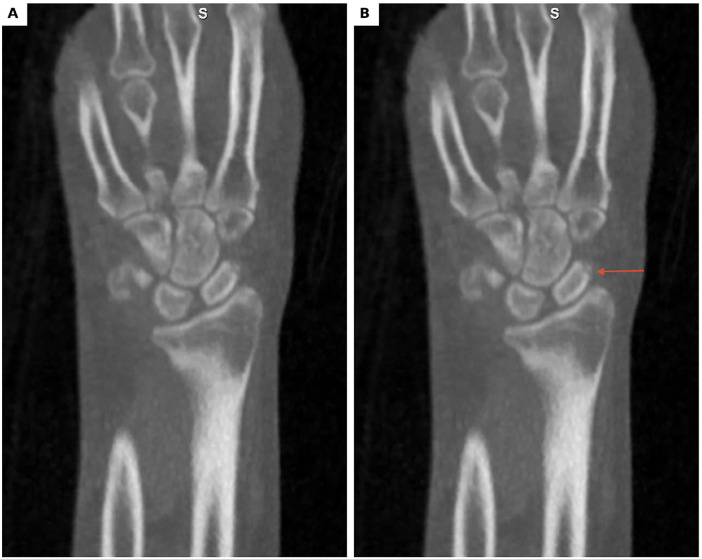
Computed tomography of the right wrist demonstrating the non-displaced scaphoid fracture, which was not visible on plain radiographs obtained at the presentation. **(A)** Coronal CT slice of the right wrist without annotation. **(B)** The same coronal projection with a non-displaced scaphoid fracture is indicated by the red arrow. The shortened fourth metacarpal is again appreciable in these images.

Notably, the patient reported no prior history of functional limitations or discomfort related to her bilateral congenital shortening of the fourth metacarpal prior to these injuries. She denied any prior hand trauma, and imaging revealed no degenerative changes in the bilateral hands. Upon examination after recovery, a subtle flattening of the dorsal contour of the fourth metacarpophalangeal joints was observed bilaterally, resulting in the classic “absent knuckle sign” (Figure 3, Supplementary Video 1). Bilateral hand radiography further revealed symmetric shortening of the fourth metacarpals (Figure 4). She reported a history of competitive athletic activity, playing softball at the high school level and internationally as part of her national team.

**Figure 3:**
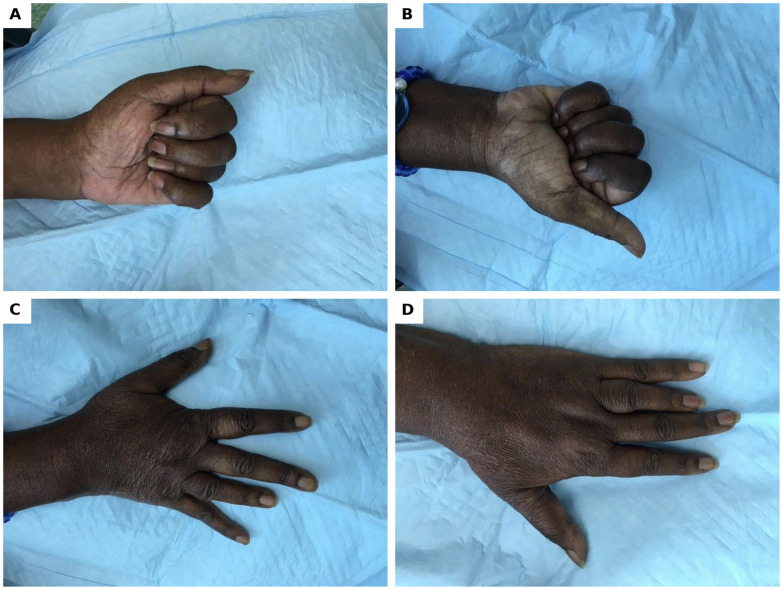
Clinical photographs of bilateral hands. **(A,B)** Closed-fist position of each hand demonstrating flattening of the dorsal contour at the fourth metacarpophalangeal joint, producing the absent knuckle sign, characteristic of fourth metacarpal shortening; full digital flexion is preserved. **(C,D)** Dorsal view of each hand with the fingers fully extended, demonstrating flattening of the fourth metacarpal head prominence and a relatively shortened ring finger compared to the adjacent digits.

**Figure 4:**
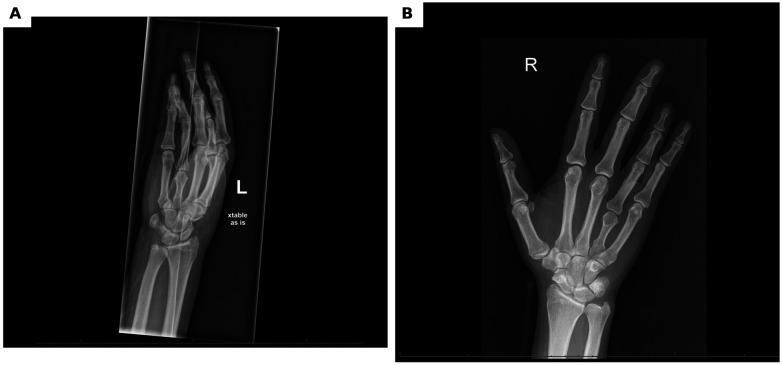
Bilateral hand radiographs demonstrating symmetric congenital shortening of the fourth metacarpals with preserved cortical margins and joint spaces. **(A)** Left hand radiograph (cross-table oblique projection); the imaging technique on this view is suboptimal, but the fourth metacarpal shortening remains evident. **(B)** Right-hand posteroanterior radiograph clearly demonstrating the hypoplastic fourth metacarpal diaphysis with normal head and base morphology. The fractures are not annotated on these radiographs because both were characterized on CT (see Figures 1, 2).

### 2.1 Bone health assessment

Formal bone health evaluation, including dual-energy X-ray absorptiometry and assessment of vitamin D, calcium, phosphate, and parathyroid hormone levels, was not performed during the episodes of care; these fractures are not strictly categorized as fragility fractures. The patient was taking vitamin D 5,000 IU daily at the time of the second injury. Osteoporosis-directed pharmacotherapy and structured secondary fracture prevention were not documented in this study. Given the patient's age, two separate low-energy wrist fractures, and elevated risk for future falls, the absence of a formal bone health assessment is acknowledged as a limitation. In patients with similar conditions, it is advisable to evaluate bone mineral density for the prevention of secondary fractures.

## 3 Timeline

A summary of the clinical timeline is presented in Table 1.

**Table 1:** Timeline of clinical events, diagnostic findings, and outcomes.

Time point	Event	Findings	Intervention/outcome
2010	Archived radiographs of the right hand obtained	Shortened fourth metacarpal of the right hand (retrospectively identified); left hand not imaged at this time	No intervention; right-hand baseline established retrospectively
2017	First fall onto left wrist; ED evaluation with radiographs and CT of the left hand and wrist	Non-displaced triquetral fracture on CT; shortened left fourth metacarpal noted incidentally	Wrist-control splint for six weeks; occupational therapy; complete fracture union; full range of motion (ROM) restored at follow-up
2018	Second fall onto right wrist; ED evaluation with radiographs and CT of the right hand and wrist	Plain radiographs initially negative; CT demonstrated non-displaced scaphoid fracture; identical right fourth metacarpal shortening; bilateral involvement recognized	Cast immobilization for three months; occupational therapy; follow-up CT at 4 months confirmed bony union; full pain-free ROM bilaterally at five months
Follow-up	Outpatient clinical review	Full ROM, absent knuckle sign bilaterally; no degenerative changes; stable radiographic appearance over >10 years	Diagnosis: isolated bilateral congenital type E brachydactyly; no further treatment required

## 4 Diagnostic assessment, therapeutic intervention, follow-up, and outcomes

The diagnostic workup at both ED presentations included plain radiography and CT imaging. Left wrist evaluation in 2017 revealed a non-displaced triquetral fracture, confirmed on CT (Figure 1), with an incidentally noted shortened fourth metacarpal. The right wrist evaluation following the second fall in 2018 was more nuanced. Plain radiographs of the right wrist and hand obtained in the ED, without a dedicated scaphoid view, were initially negative for fracture. A CT scan subsequently ordered by another orthopedic surgeon identified an acute non-displaced scaphoid fracture (Figure 2) and showed identical fourth metacarpal shortening, at which point bilateral congenital involvement was recognized. Comparison with archived radiographs of the right hand from 2010 demonstrated a stable appearance of the fourth metacarpal for more than a decade, further supporting a congenital etiology. Radiographic and clinical findings were consistent with isolated congenital type E brachydactyly. Three-dimensional CT reconstructions further illustrated the symmetric diaphyseal shortening of the fourth metacarpal (Figures 1C, D). Due to the patient's age, the absence of parathyroid or other endocrine diseases, and a long history of normal hand function, no further syndromic evaluation was conducted.

### 4.1 Therapeutic intervention and rehabilitation

Conservative management was used for both fractures. The left triquetral fracture was treated with a wrist-control splint for 6 weeks. The patient was referred for formal occupational therapy, with a protocol focused on simple range-of-motion exercises for the fingers and wrist; no adjunctive modalities, such as shockwave therapy, were employed. Recovery was defined by clinical examination and the patient’s reported return to baseline activities, both of which were achieved by the 6-week follow-up, with complete fracture union and full digital range of motion.

The right scaphoid fracture was managed with cast immobilization for three months, followed by formal occupational therapy consisting of range-of-motion exercises of the fingers and wrist, again without adjunctive modalities. A follow-up CT scan obtained 4 months after the injury confirmed bony union of the scaphoid fracture, and a clinical examination at 5 months post-injury demonstrated a full, pain-free range of motion bilaterally. At the final follow-up, the patient demonstrated a full, pain-free range of motion bilaterally.

## 5 Discussion

Brachydactyly type E encompasses the congenital shortening of one or more metacarpals resulting from the premature closure or underdevelopment of the growth plate. Genetically, it has been associated with pathogenic variants affecting *PTHLH* (15) and mutations in *GNAS* and *EXT1* (16). Among these, isolated involvement of the fourth metacarpal is the most frequent and is often an incidental finding (5, 6, 17). This condition may occur sporadically or in association with endocrine or genetic syndromes (1–3). Bilateral isolated cases are rare.

Radiographically, congenital fourth metacarpal shortening manifests as a hypoplastic diaphysis, while the head and base maintain normal morphology, and the metacarpophalangeal and carpometacarpal joints are preserved. In our patient, identical shortening on both sides, preserved cortical outlines, absence of degenerative changes, and stable radiographic appearance over more than a decade strongly support a developmental rather than an acquired etiology.

The two wrist fractures in this patient were low-energy injuries sustained after separate falls and are best understood as the result of fall mechanics and host factors rather than congenital fourth metacarpal shortening. The shortening involves the fourth metacarpal diaphysis and does not alter carpal anatomy, and there is no biomechanical basis by which it would predispose to triquetral or scaphoid fractures. Comorbid type II diabetes mellitus, peripheral artery disease, and prior lower extremity amputation are recognized contributors to impaired balance and altered gait, and the patient's age raises the possibility of reduced bone strength. The congenital variant was an incidental radiographic finding identified during the trauma workup and was not implicated in either injury.

### 5.1 Comparison with similar and contrasting cases

Previous case reports provide useful context. Kirkos (11) described bilateral involvement of the fourth and fifth metacarpals and metatarsals, while Nkosi et al. (12) reported bilateral symmetric brachymetacarpia of the ring fingers. Aski et al. (10) documented bilateral idiopathic shortening in an adult who was thoroughly investigated to exclude syndromic associations, and Tazi et al. (13) described bilateral shortening of the fifth metacarpals in a 60-year-old case. These reports collectively establish that isolated congenital metacarpal anomalies can present in adults as incidental and benign findings. However, none of the studies examined functional outcomes beyond the absence of symptoms, nor did any report involvement in competitive athletics.

Our case adds a new dimension to this knowledge. The fourth ray minimally contributes to the overall grip arc, and compensatory hyperextension of the metacarpophalangeal joint often masks the deformity. Previous reports have documented functional preservation only in the context of routine daily activities. Our patient competed in softball at both the high school level and internationally as part of her national team, an activity requiring repetitive forceful gripping for batting, throwing mechanics, and rapid glove-to-hand ball transfers. Her participation at this level without any self-reported functional limitation provides supportive evidence that this variant imposes no clinically apparent functional ceiling, although this conclusion is based on retrospective patient reports rather than objective measurements.

### 5.2 Strengths and limitations

The principal strength of this case is the documentation of sustained competitive athletic participation in the setting of bilateral congenital shortening of the fourth metacarpal, a functional outcome not previously reported. Moreover, archived radiographs spanning more than a decade provided the longest documented radiographic follow-up for this variant in an adult patient, confirming the non-progressive nature of the condition.

This study has several limitations. The functional history was retrospective and based on patient self-reports, and the athletic history was not independently verified. No objective functional assessment was performed; grip and pinch strength dynamometry, wrist and finger range-of-motion measurements, and validated outcome instruments such as the Disabilities of the Arm, Shoulder and Hand (DASH) questionnaire or QuickDASH were not obtained. Consequently, statements regarding preserved function should be interpreted as the absence of self-reported limitations rather than objectively confirmed normal function. Formal bone health evaluation and secondary fracture prevention were not documented (see Section 2.1). Finally, the single-patient design limited the generalizability of the results.

### 5.3 Take-away lessons

This case demonstrates that bilateral congenital shortening of the fourth metacarpal can remain clinically silent throughout a lifetime of high-demand hand use, including competitive softball at the national team level. Recognition of this radiographic appearance can prevent misinterpretation as traumatic shortening or growth plate injury and reassure both the patient and clinician. Conservative management with reassurance is appropriate for asymptomatic patients. Metacarpal lengthening procedures (14, 15) entail risks of stiffness, non-union, and infection that outweigh the potential benefits in asymptomatic adults and should be reserved for select cosmetic indications.

## 6 Patient perspective

The patient reported no prior history of functional limitations or discomfort related to bilateral shortening of the fourth metacarpal. She noted that her hands had never limited her performance in competitive softball at either the high school or international level or in any other manual activity. Her power of attorney consented to the release of her case in the hope that it may help other individuals with this finding avoid unnecessary worry or treatment in the future.

## Data Availability

The original contributions presented in this study are included in the article/Supplementary Material, Further inquiries can be directed to the corresponding authors.

## Supplementary material

Supplementary Video S1 Short video clip demonstrating the patient exhibiting a full range of motion of both hands as she opens and closes her fists, illustrating preserved digital flexion and extension despite bilateral fourth metacarpal shortening.
